# Supplementary figures and images for: A distribution model for Glossina brevipalpis and Glossina austeni in Southern Mozambique, Eswatini and South Africa for enhanced area-wide integrated pest management approaches
Source: PLoS Negl Trop Dis. 2021 Nov 29;15(11):e0009989. doi: 10.1371/journal.pntd.0009989 (PMC8659649; doi:10.1371/journal.pntd.0009989)

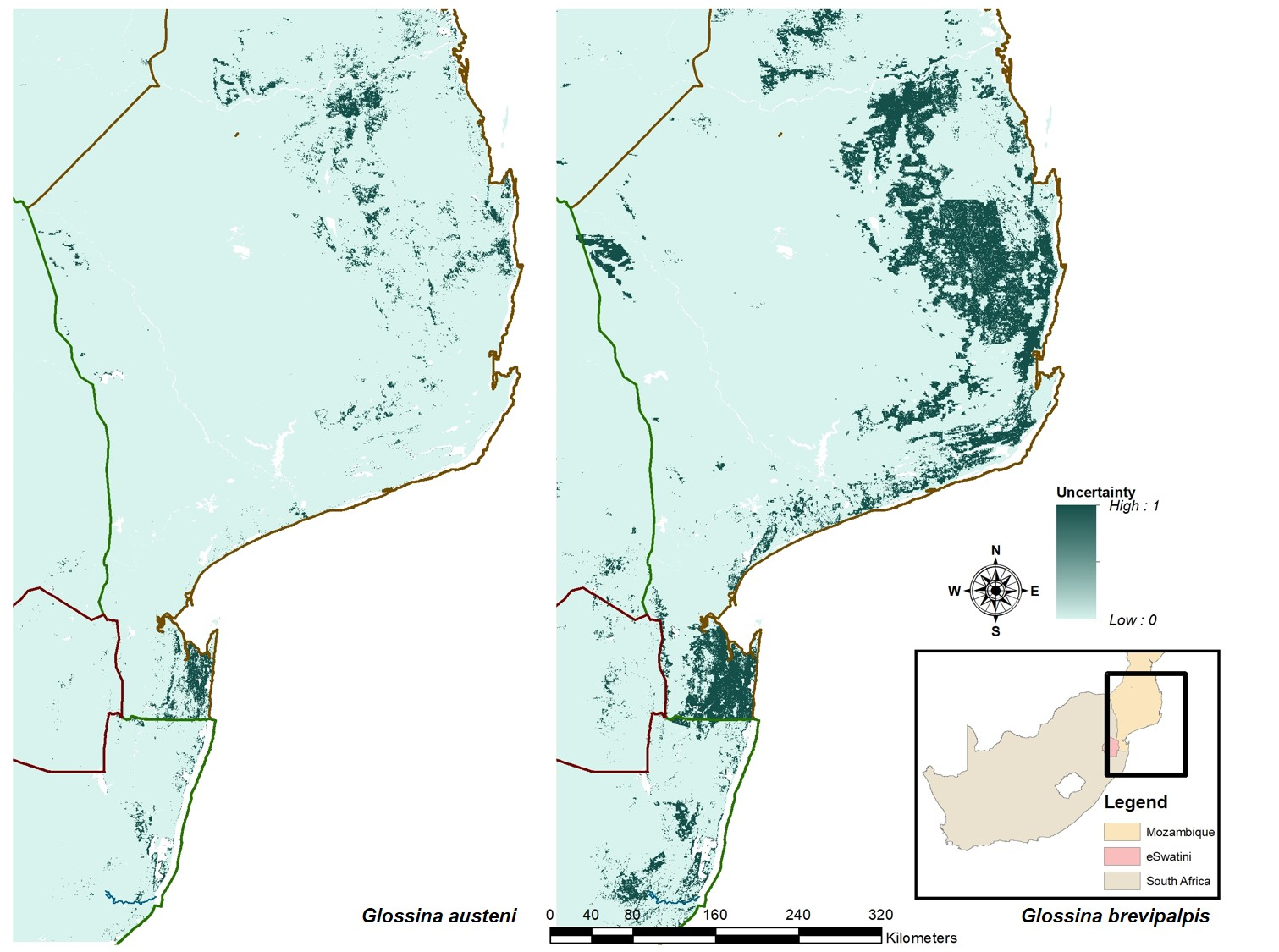

Supplement: S1 Fig — (TIF) [file pntd.0009989.s001.tif]
