# Supplementary material for: A distribution model for Glossina brevipalpis and Glossina austeni in Southern Mozambique, Eswatini and South Africa for enhanced area-wide integrated pest management approaches
Source: PLoS Negl Trop Dis. 2021 Nov 29;15(11):e0009989. doi: 10.1371/journal.pntd.0009989 (PMC8659649; doi:10.1371/journal.pntd.0009989)
Supplement: Attachment — Submitted filename: Response to reviewers_final.docx [file pntd.0009989.s002.docx]

Response to reviewers

We are grateful for the effort of the reviewers to improve the quality of the manuscript.

All suggested changes as well as some additional changes were made in the manuscript in track changes. Additionally, please see response to the comments below.

Reviewer #2: There is insufficient detail provided on a number of aspects of the methodology.

-	The objective of the study should be stated more clearly at the end of the introduction.

**Response: The objective of this study was added at the end of the introduction.**

Study site descriptions and tsetse data collection section:

-	Have any of these data been published before? Reference if so.

**Response: It is stated that “existing entomological survey data” was used to develop the models. It is now stated that the data were not published before.**

-	How were the locations of tsetse trapping sites chosen given this is important for MaxEnt to produce robust results?

**Response: the following was added to the Tsetse data collection section “Trapping sites were selected from areas that had known presence of tsetse or were predicted as positive by the existing probability of presence model”.**

-	Line 196-197 Does this mean that some traps were in the same location for 2313 days? Did this affect trap catches, i.e. you may expect to see a decline in population over time?

**Response: “2313” is an error. The trapping days were corrected to “244”. It was added that “Considering the number of traps deploy per site and the number of trapping days it is envisage that the influence on AD was minimal.”**

-	It isn’t stated what type of data were collected on the tsetse trapped. Presumably they were identified morphologically, sexed and counted?

**Response: the following was added to the Tsetse data collection section “The collected tsetse flies were counted, identified morphologically to species level and sexed.”**

-	Line 228 ‘ANOVA was used to differentiate between the mean tsetse fly AD’. Do you mean it was used to compare AD between different locations? How were the confidence intervals calculated on the mean ADs?

**Response: The sentence was restructured for clarity.**

-	Line 229 Apparent density needs to be defined, and it should be explained how it was calculated

**Response: the following was added to the tsetse data collection section “The number of each species collected over this period was counted and results expressed as apparent density (AD), i.e. the number of flies per trap per day.”**

-	From line 233 The process for generating pseudoabsences isn’t very clear. How exactly were they generated and how many were used?

From line 233 How were repeat samples from the same location used in the analysis? The filter process isn’t explained very clearly – were repeat measures subsumed into a single presence point by this filtering? Was the season of tsetse collection considered in the analysis?

**Response:** **Pseudo-absences were generated randomly in the grid according the a kernel based on sampling effort. The more we sampled an area the more pseudo-absence we generated and areas less sampled will have less. The kernel was built using spatial point pattern analysis using all traps information we could get on the study area. In a second step, we picked one pseudo-absences per raster cell (approx. 500 x 500 m). Finally, we removed all pseudo-absences that felt into the 2 km radius buffer around presence data, based on data on flight range of these species. The process was repeated several times and stored for the analysis. The section “Spatial filtering and generation of pseudo-absence was expanded with more detail.**

-	Line 253 – Field surveys are mentioned here but not described. What field data were collected and how were the data used?

**Response: the following was added to the Remote sensing data section “The field records comprise of 1092 photographs that were taken at locations within the known tsetse distribution area of SA, SM and SW. The photographs were used, based on the international nomenclature, to identify 11 main types of vegetation units that were relevant to tsetse habitat mapping.”**

-	Line 302 What exactly is meant by ‘to assess the effect of pseudo-absence’

**Response: A sentence was included that states “****The effect of pseudo-absences was assessed by repeating the process to generate them several times and monitor variability and stability in model quality metrics.”**

-	Line 303 Two independent datasets are mentioned here but not described. In the abstract one independent dataset is mentioned. Details of these datasets should be included.

**Response: The reference to “two independent datasets” was a mistake. The sentence was changed to indicate a subset of the original dataset as indicated in the “Tsetse occurrence and density” section was used.**

-	The method used to measure uncertainty is not described.

**Response: A sentence on how uncertainty was measured was added** **“Uncertainty was measured using the coefficient of variation from the predictions”.**

Reviewer #2: Mostly the results are clearly described although some things had not been included in the methods. Figures are clear and nicely presented.

**Response: Additional descriptions were added to the methods section.**

Line 359-362 This finding regarding which factors influence distribution is obviously important. Whilst it is clearly stated here that human and cattle density have a negative association with occurrence, in other places e.g. line 440, 477, abstract, it isn’t made clear that it is a negative relationship.

**Response: This negative association was highlighted again in the discussion section.**

Lines 417-421 Is extrapolating beyond the limits of data collection justified? This part should also be described in the methods.

**Response: As indicated in the maximum entropy ecological niche model section: ”When the model was extended northwards to the Gaza and Inhambane Provinces of Mozambique the uncertainty increased. However, the prediction values along the coastline in the Inhambane Province retained relative high certainty”.** **Extrapolating beyond the** **data collection limits is justified as it gives a starting point to where collection efforts can be prioritised in the unknown area, which will reduce effort and cost in the initial stages of the entomological baseline data collection.**

**A sentence was added to the M & M section: “Based on the predictive quality of the MaxEnt models develop, and keeping the degree of uncertainty in mind, the potential distribution of the two species were determined by extrapolation of the model to adjoining areas outside the collection limits.”**

**Reviewer #2: In general the relevance of the findings is discussed and mostly valid conclusions are reached. The limitations of the approach chosen are not addressed sufficiently.**

**Response: A section was added in the discussion to address** **the choice of the model selected.**

- Line 436 ‘whilst incorporating the ecology of both species’ I’m not convinced this approach does incorporate the ecology – remove or be more specific

**Response:** **“using statistical methods whilst incorporating the ecology of both species” was changed to “using statistical methods whilst incorporating** **what is known of the ecology, e.g., host and habitat preferences, of both species.**

- Line 440 should say presence of cattle, not presence of host animals, since you didn’t assess wildlife hosts.

**Response: although “wildlife hosts” were not directly assessed the presence of wildlife hosts in all the protected areas did play a role in the positive association observed for *G. brevipalpis.* The sentence was change to “In agreement with previous entomological surveys in the affected area in north-eastern KZN, climate, vegetation and presence of protected areas and cattle were the key regulators that determined the presence and abundance of *G. brevipalpis* *and G. austeni*”**

- Lines 476-486 The logic isn’t totally clear here but I think you are suggesting that the negative relationship between cattle density and tsetse presence is most likely due to dipping of cattle in farming areas. Whilst I agree it is plausible, how can you differentiate this hypothesis from there being less appropriate tsetse habitat in farming areas?

**Response: The sentence was restructured as: “For both species, cattle densities, irrespective of otherwise suitable habitat, showed a negative association with occurrence. This apparent contradictive observation may be the result of the fact that cattle are used currently used as live bait for tsetse control in north eastern KZN.”**

- Line 496 It would be more accurate to say the presence of buffalo and other wildlife hosts, since there are many species that can carry T. congolense and/or T. vivax.

**Response: The sentence was changed as suggested.**

Reviewer #2: - Line 51 should be ‘practices’

**Response: Change made as suggested.**

-	Reference 14 does not support the statement where it is used, since it doesn’t include any assessment of vector competence. Reference 15 is appropriate but the sentence should read ‘more competent for T. congolense’, since it was only this species that was assessed.

**Response: Reference 14 was replaced with a more appropriate reference and the sentence was changed as suggested.**

-	Line 95-96 check grammar

**Response: Grammar was corrected**

-	What is already known about the habitat preferences or predictors of brevipalpis and austeni presence should be stated in the intro. There is literature on this that is cited later, e.g. ref 18, 28, 39 but this should be mentioned in the introduction and the specific novelty of this study identified.

**Response: It is indeed stated in the introduction: “Localised surveys and available distribution prediction models for South Africa showed that, on a micro-ecological scale, G. brevipalpis and G. austeni are confined to pockets of dense vegetation in north-eastern KZN [16–19].**

-	Lines 128-129 The Leak book, in its entirety, is an odd reference to use here. The more recent and specific literature on this topic should be cited, e.g. Hargrove et al. 2012. Also applies to line 541

**Response: The “Leak” reference was replaced with the more appropriate “Hargrove” reference as suggested.**

-	Lines 130-133 States that an AW-IPM strategy that includes an SIT component is required. Whilst an AW-IPW is clearly required, SIT is not the only option and the wording of this sentence should be edited to reflect that.

**Response: The sentence was restructured, and more information provided.**

-	Line 133-135 Reference 22 should come at the end of the first part of the sentence.

**Response: The reference was moved as suggested.**

-	Line 153-155 This sentence is rather vague and it isn’t clear whether it is stating an objective of the study or simply commenting on the potential use of the distribution maps in the future. Suggest remove and instead add a sentence clearly stating the objective of this study.

**Response: The objective of this study was added at the end of the introduction.**

-	Line 193 – does this mean there were seven sachets per trap?

**Response: Yes, this is correct, the word “each” has been inserted.**

-	Line 260-261 Gridded livestock data – the version should be stated and appropriate reference included. World Database on Protected Areas should be referenced appropriately.

**Response: Change was made as suggested.**

-	Lines 316 to 353 I wonder if this whole section would be better presented in a table or a figure.

**Response: Although a Table/figure may allow a better representation of the data the relevant data points will still need to be extracted and mentioned in the text for interpretation. The purpose of the text is to highlight differences and variation between sites. Considering the number of Tables and Figures already presented, additional Tables may be superfluous.**

Materials and methods

Line 165: add “,” after “In Eswatini”

**Response: Change was made as suggested.**

Lines 170 – 172: The fig 1 shows tsetse apparent densities and could be announced in the results section. May be it will be better to designed a figure which showing only the study area without the tsetse densities.

**Response: Considering the number of figures already depicting the sites an additional figure may be superfluous.**

Line 190: “are” must be added after “incorporated”?

**Response: Change was made as suggested.**

Lines 196 – 199: the authors indicated that “In total 160 H-traps were deployed for a minimum of 19 and a maximum of 2313 trapping days. Flies were collected from the traps and the traps serviced (clearing of vegetation, odour replacement, replace the traps when the colours are faded etc.) every 14 days.”

Generally, for just tsetse sampling for presence/absence and abundance evaluation, around 3 days of traps deployment is enough but in this study, the trap was kept until 2313 trapping days. It seem to be a tsetse control program where traps were deployed for monitoring, the authors must notify that in the methodology section.

**Response: The number “2313” is an error. The trapping days were corrected to “244”. The number of traps at only specific sites that collected for 244 days were not able to significantly negatively impact the tsetse abundance. It is also now mentioned that the data were obtained from routine, and yet unpublished, entomological surveys using H-traps.**

Line 229: what means “AD”? …… add “apparent density (AD)”

**Response: the following was added to the Tsetse data collection section “The number of each species collected over this period was counted and results expressed as apparent density (AD), i.e. the number of flies per trap per day.”**

Line 231: add “is” before “< 0.05”

**Response: The sentence was changed.**

Line 299: add “,” after “derived index”

**Response: Change was made as suggested.**

Line 317: put space after “±” in the sentence “(2.14 ±5.4) in MP. However, the lower mean AD (0.08 ±0.2)”. The same correction is necessary in the rest of the result section.

**Response: The section was corrected as suggested.**

Line 321: delete the space between “(x” in the sentence “followed by shrub savannah (x AD of 6.46 ±7.9)”. The same correction is necessary in the rest of the result section.

**Response: The section was corrected as suggested.**

Line 336: add “,” after “In Eswatini”

**Response: Change was made as suggested.**

Line 544: add “,” after “In 2007”

**Response: Change was made as suggested.**

Line 600: replace “van” by “Van”

**Response: Change was made as suggested.**

Reviewer #2: This paper presents a distribution model for G. austeni and G. brevipalpis for the tsetse belt that covers parts of South Africa, Mozambique and Eswatini. The novelty of the work is that it uses data collected in all three countries to look at area-wide distribution to assist development of regional tsetse control plans and will provide a valuable tool for this purpose.

Weaknesses – The study does not address a new question or describe a novel approach, although it is a useful example of using regional distribution modelling to inform control. The MaxEnt approach used, whilst commonly used for predicting the probability of presence of vectors, uses presence only data inputs. Other approaches could have been considered to make the most of the abundance data collected. For several aspects of the methodology, the level of detail provided is not sufficient to fully understand what has been done (see specific comments). The limitations of the approach are not discussed. The clarity of writing could be improved in places (see specific comments).

**Response: The level of detail in the methodology section has been improved.**

Reviewer #3: Comments on PNTD-D-21-00569 This paper uses entomological data on captures of the two main tsetse vectors of african trypanosomes in the southern Africa region, in order to develop prediction maps and identify habitat suitability for tsetse presence/absence. These information are key, if not essential to the development of tsetse control programmes. The ms is of very good quality, and is of help and relevance.

Majors comments

The authors should add information on the contraint due to AAT which is absent here : what are the prevalences ? costs associated ? etc., any information would help. The reader understands that the initiative comes from South Africa where there are control programmes on-going, but how is it preceived in Mozambique and Eswatini ? I appreciate this goes a bit beyond the purely scientific aspcet of the paper, but it would certainly help also.

**Response: A paragraph was added to the Introduction**

Minor comments

Introduction, line 76 : « and they infest about 10 million km² in sub-Saharan Africa » : do you really believe this ? ref 2 is not appropriate here, and may be certainly cited elsewhere in the text. But you may rather write « are reported to infest », or include some nuance here, as you are well placed to know what you are speaking about…

**Response: Reference 2 was removed and sentence was changed as suggested.**

Introduction, line 79-80 : please include a ref for HAT, since you mention two diseases, but only give ref for the animal one. In addition, speaking about the human disease, you may include an impact more related to health than to economy…

**Response: Information and a reference on HAT was added as suggested: “Human African Trypanosomosis, a fatal disease if left untreated, although absent in southern Africa, occurs regularly in some regions of sub-Saharan Africa with the population at risk being about 70 million in 36 countries (Simarro et al. 2012)”**

Introduction, line 89 : having written Trypanosoma in full a first time here, you may replace it by « T. » on all other occurrences (l. 89, 90, 92 ? etc).

**Response: Change was made as suggested. The technical editor can decide if species names need to abbreviated if at the beginning of a sentence.**

Introduction, lines 130-132 : « The establishment of a tsetse-free zone in southern Africa would be a more sustainable solution to the nagana problem, but would require an AW-IPM strategy that includes an SIT component ». Although the beginning of your statement is correct, please end it by something less subjective, and more appropriate. I don’t think SIT is the only tool that can lead to tsetse eradication, especially in this southern area of tsetse distribution where tsetse have also been eradicated from neighbouring countries by aerial spraying, correct me if I am wrong ?

**Response:** **The sentence was restructured, and more information provided.**

Discussion, lines 428-430 : « A sound understanding of the potential distribution of targeted tsetse fly species will be essential for the successful implementation of an AW-IPM programme, especially those that include an SIT component ». Not only I agree here, but please be more global because this « sound understanding » is essential, if not mandatory, for ANY control programme, again yes for those which include an SIT component, but not only for them…

**Response: The sentence was restructured as “A sound understanding of the potential distribution of targeted tsetse fly species will be essential for the successful implementation of any AW-IPM programme, e.g., it will establish the area over which sterilized males need to be released in programmes that include an SIT component.”**

Discussion lines 506-508 : « Therefore, the current model predicted a smaller tsetse fly infested area than previous models. This may be due to model refinement, changes in the habitat as well as the apparent success of the current tsetse control actions in the area. » may be you could expand a bit here, to help the reader’s understanding. Why those « changes in habitat », what would be the cause, e.g. human growth, habitat destruction, why not even climate change, I am sure you know better than me.

**Response: A sentence was added. “The reason for this is not clear but it can be speculated that this may be due to model refinement, changes in the habitat (e.g., bush clearing for agricultural purposes) as well as the apparent success of the current tsetse control actions in the area. Considering the sensitivity of tsetse to environmental influence this can even be indictive of the result of climate change in the area. A more intensive study will be needed to pinpoint the exact reason for the predicted a smaller tsetse fly-infested area.”**

Discussion line 510-511 : « an area suspected to be free of tsetse following a reduction in cattle and wildlife numbers », the reader may want to understand why is that so ? I mean why a reduction in cattle and wildlife numbers?

**Response: It was added that this may have been due to “due to human settlement and population expansion”**
